# Corrigendum

**DOI:** 10.1080/13880209.2016.1240285

**Published:** 2016-12-08

Guo Y, Wang S, Wang Y, Zhu T. 2016. Silymarin improved diet-induced liver damage and insulin resistance by decreasing inflammation in mice, Pharm Biol.

http://dx.doi.org/10.1080/13880209.2016.1199042

When the above article was first published online, there was a spelling error in the first author’s name. This has now been corrected in the online and print versions.

